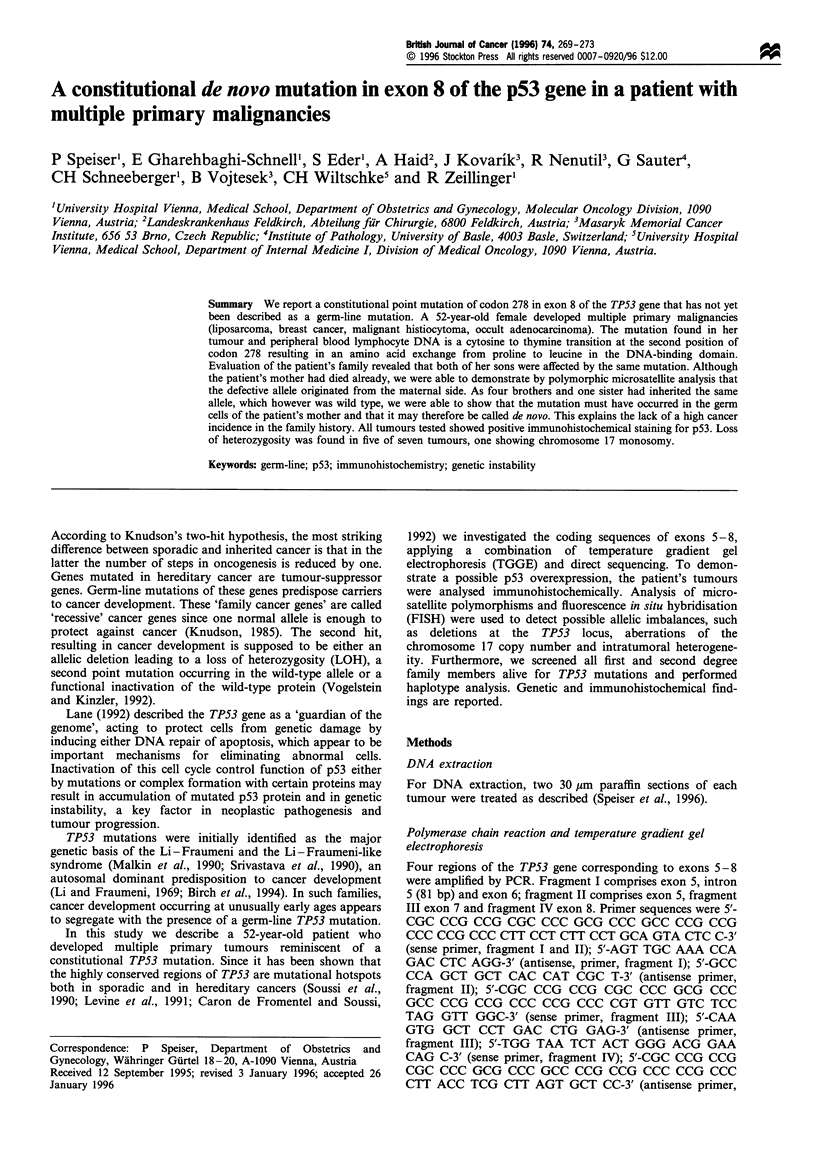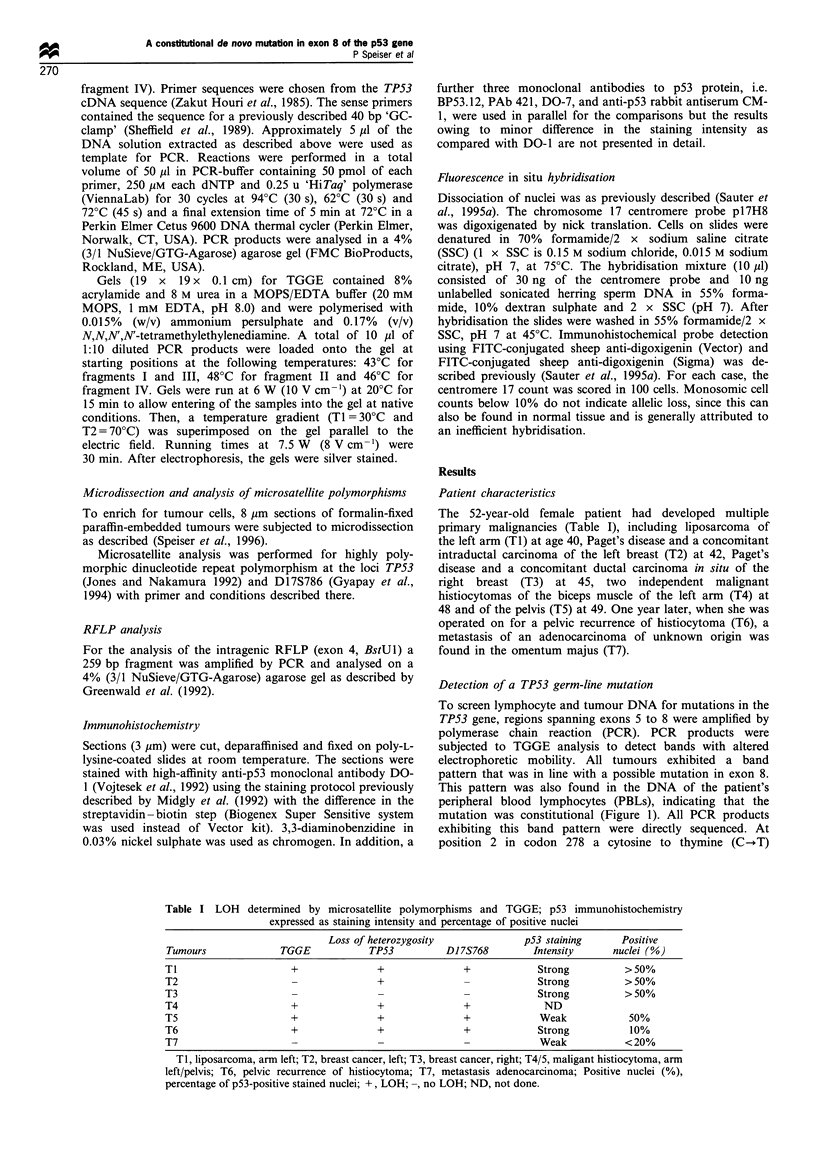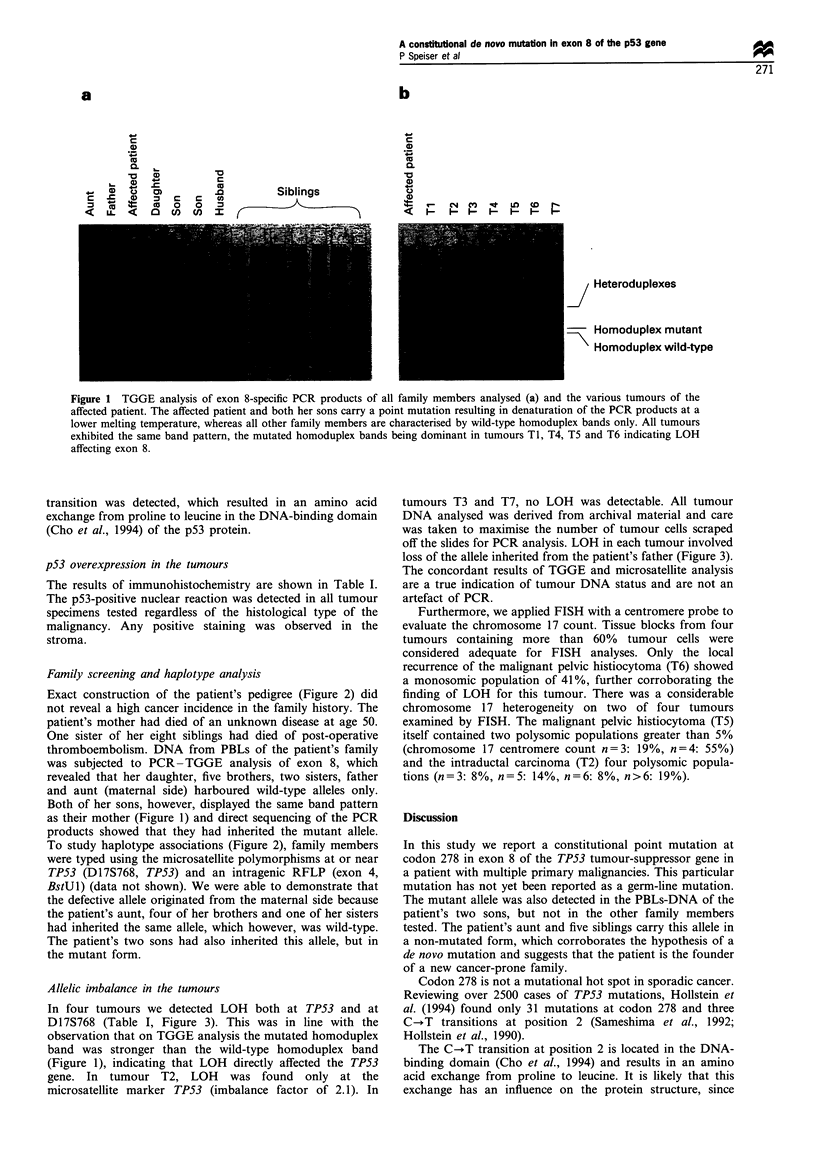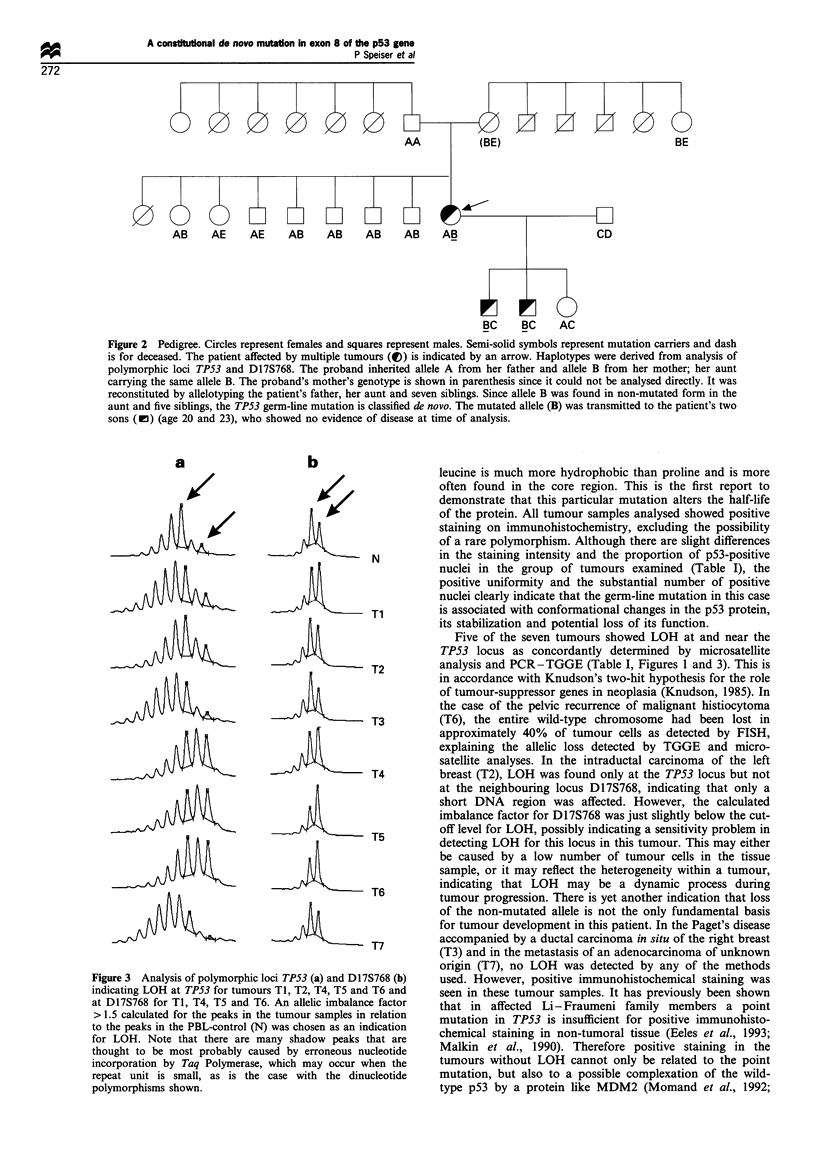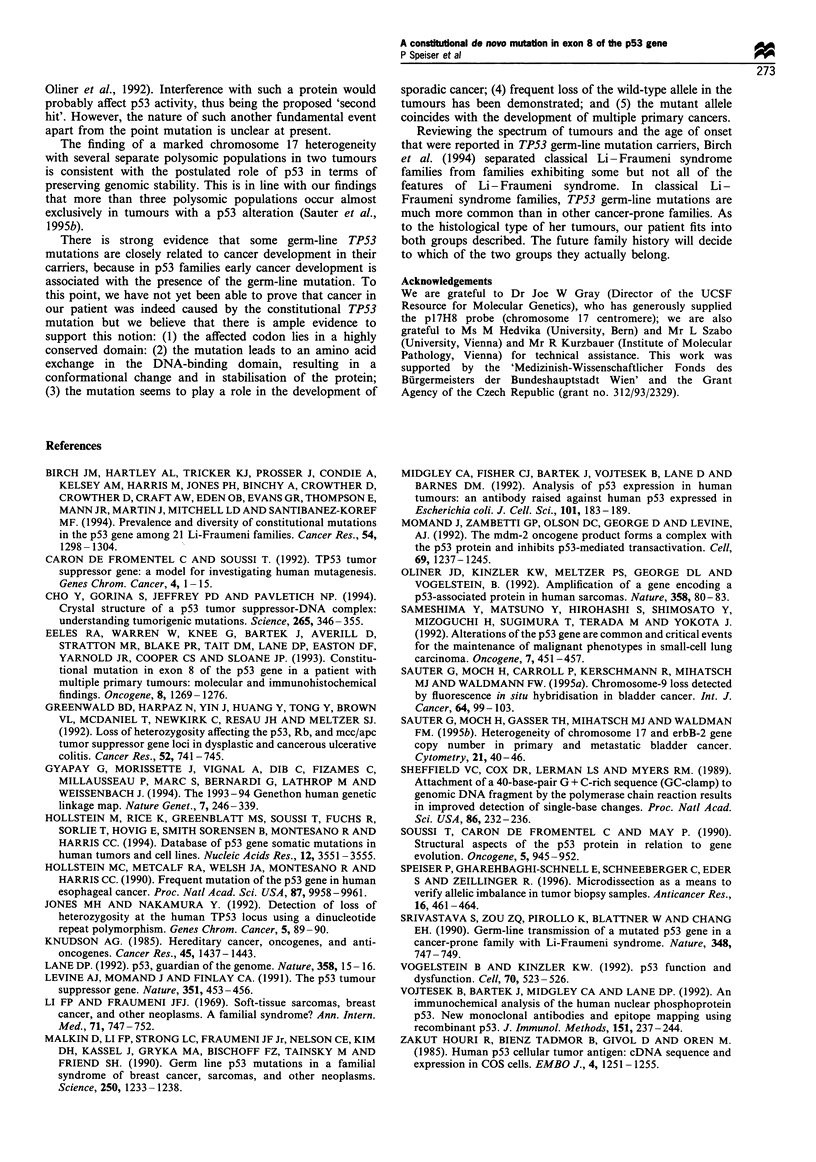# A constitutional de novo mutation in exon 8 of the p53 gene in a patient with multiple primary malignancies.

**DOI:** 10.1038/bjc.1996.350

**Published:** 1996-07

**Authors:** P. Speiser, E. Gharehbaghi-Schnell, S. Eder, A. Haid, J. Kovarík, R. Nenutil, G. Sauter, C. H. Schneeberger, B. Vojtesek, C. H. Wiltschke, R. Zeillinger

**Affiliations:** University Hospital Vienna, Medical School, Department of Obstetrics and Gynecology, Austria.

## Abstract

**Images:**


British Journal of Cancer (1996) 74, 269-273
© 1996 Stockton Press All rights reserved 0007-0920/96 $12.00

### A constitutional *de novo* mutation in exon 8 of the p53 gene in a patient with multiple primary malignancies

P Speiser[1], E Gharehbaghi-Schnell[1], S Eder[1], A Haid[2], J Kovarík[3], R Nenutil[3], G Sauter[4], CH Schneeberger[1], B Vojtesek[3], CH Wiltschke[5] and R Zeillinger[1]

[1]*University Hospital Vienna, Medical School, Department of Obstetrics and Gynecology, Molecular Oncology Division, 1090 Vienna, Austria;* [2]*Landeskrankenhaus Feldkirch, Abteilung für Chirurgie, 6800 Feldkirch, Austria;* [3]*Masaryk Memorial Cancer Institute, 656 53 Brno, Czech Republic;* [4]*Institute of Pathology, University of Basle, 4003 Basle, Switzerland;* [5]*University Hospital Vienna, Medical School, Department of Internal Medicine I, Division of Medical Oncology, 1090 Vienna, Austria.*

**Summary** We report a constitutional point mutation of codon 278 in exon 8 of the *TP53* gene that has not yet been described as a germ-line mutation. A 52-year-old female developed multiple primary malignancies (liposarcoma, breast cancer, malignant histiocytoma, occult adenocarcinoma). The mutation found in her tumour and peripheral blood lymphocyte DNA is a cytosine to thymine transition at the second position of codon 278 resulting in an amino acid exchange from proline to leucine in the DNA-binding domain. Evaluation of the patient's family revealed that both of her sons were affected by the same mutation. Although the patient's mother had died already, we were able to demonstrate by polymorphic microsatellite analysis that the defective allele originated from the maternal side. As four brothers and one sister had inherited the same allele, which however was wild type, we were able to show that the mutation must have occurred in the germ cells of the patient's mother and that it may therefore be called *de novo*. This explains the lack of a high cancer incidence in the family history. All tumours tested showed positive immunohistochemical staining for p53. Loss of heterozygosity was found in five of seven tumours, one showing chromosome 17 monosomy.

**Keywords:** germ-line; p53; immunohistochemistry; genetic instability

According to Knudson's two-hit hypothesis, the most striking difference between sporadic and inherited cancer is that in the latter the number of steps in oncogenesis is reduced by one. Genes mutated in hereditary cancer are tumour-suppressor genes. Germ-line mutations of these genes predispose carriers to cancer development. These 'family cancer genes' are called 'recessive' cancer genes since one normal allele is enough to protect against cancer (Knudson, 1985). The second hit, resulting in cancer development is supposed to be either an allelic deletion leading to a loss of heterozygosity (LOH), a second point mutation occurring in the wild-type allele or a functional inactivation of the wild-type protein (Vogelstein and Kinzler, 1992).

Lane (1992) described the *TP53* gene as a 'guardian of the genome', acting to protect cells from genetic damage by inducing either DNA repair of apoptosis, which appear to be important mechanisms for eliminating abnormal cells. Inactivation of this cell cycle control function of p53 either by mutations or complex formation with certain proteins may result in accumulation of mutated p53 protein and in genetic instability, a key factor in neoplastic pathogenesis and tumour progression.

*TP53* mutations were initially identified as the major genetic basis of the Li-Fraumeni and the Li-Fraumeni-like syndrome (Malkin *et al.*, 1990; Srivastava *et al.*, 1990), an autosomal dominant predisposition to cancer development (Li and Fraumeni, 1969; Birch *et al.*, 1994). In such families, cancer development occurring at unusually early ages appears to segregate with the presence of a germ-line *TP53* mutation.

In this study we describe a 52-year-old patient who developed multiple primary tumours reminiscent of a constitutional *TP53* mutation. Since it has been shown that the highly conserved regions of *TP53* are mutational hotspots both in sporadic and in hereditary cancers (Soussi *et al.*, 1990; Levine *et al.*, 1991; Caron de Fromentel and Soussi, 1992) we investigated the coding sequences of exons 5-8, applying a combination of temperature gradient gel electrophoresis (TGGE) and direct sequencing. To demonstrate a possible p53 overexpression, the patient's tumours were analysed immunohistochemically. Analysis of microsatellite polymorphisms and fluorescence *in situ* hybridisation (FISH) were used to detect possible allelic imbalances, such as deletions at the *TP53* locus, aberrations of the chromosome 17 copy number and intratumoral heterogeneity. Furthermore, we screened all first and second degree family members alive for *TP53* mutations and performed haplotype analysis. Genetic and immunohistochemical findings are reported.

Correspondence: P Speiser, Department of Obstetrics and Gynecology, Währinger Gürtel 18-20, A-1090 Vienna, Austria
Received 12 September 1995; revised 3 January 1996; accepted 26 January 1996

### Methods

### *DNA extraction*

For DNA extraction, two 30 μm paraffin sections of each tumour were treated as described (Speiser *et al.*, 1996).

### *Polymerase chain reaction and temperature gradient gel electrophoresis*

Four regions of the *TP53* gene corresponding to exons 5-8 were amplified by PCR. Fragment I comprises exon 5, intron 5 (81 bp) and exon 6; fragment II comprises exon 5, fragment III exon 7 and fragment IV exon 8. Primer sequences were 5'-CGC CCG CCG CGC CCC GCG CCC GCC CCG CCG CCC CCG CCC CTT CCT CTT CCT GCA GTA CTC C-3' (sense primer, fragment I and II); 5'-AGT TGC AAA CCA GAC CTC AGG-3' (antisense, primer, fragment I); 5'-GCC CCA GCT GCT CAC CAT CGC T-3' (antisense primer, fragment II); 5'-CGC CCG CCG CGC CCC GCG CCC GCC CCG CCG CCC CCG CCC CGT GTT GTC TCC TAG GTT GGC-3' (sense primer, fragment III); 5'-CAA GTG GCT CCT GAC CTG GAG-3' (antisense primer, fragment III); 5'-TGG TAA TCT ACT GGG ACG GAA CAG C-3' (sense primer, fragment IV); 5'-CGC CCG CCG CGC CCC GCG CCC GCC CCG CCG CCC CCG CCC CTT ACC TCG CTT AGT GCT CC-3' (antisense primer, fragment IV). Primer sequences were chosen from the *TP53* cDNA sequence (Zakut Houri *et al.*, 1985). The sense primers contained the sequence for a previously described 40 bp 'GC-clamp' (Sheffield *et al.*, 1989). Approximately 5 $\mu$l of the DNA solution extracted as described above were used as template for PCR. Reactions were performed in a total volume of 50 $\mu$l in PCR-buffer containing 50 pmol of each primer, 250 $\mu$M each dNTP and 0.25 u 'Hi*Taq*' polymerase (ViennaLab) for 30 cycles at 94°C (30 s), 62°C (30 s) and 72°C (45 s) and a final extension time of 5 min at 72°C in a Perkin Elmer Cetus 9600 DNA thermal cycler (Perkin Elmer, Norwalk, CT, USA). PCR products were analysed in a 4% (3/1 NuSieve/GTG-Agarose) agarose gel (FMC BioProducts, Rockland, ME, USA).

Gels (19 × 19× 0.1 cm) for TGGE contained 8% acrylamide and 8 M urea in a MOPS/EDTA buffer (20 mM MOPS, 1 mM EDTA, pH 8.0) and were polymerised with 0.015% (w/v) ammonium persulphate and 0.17% (v/v) *N,N,N′,N′*-tetramethylethylenediamine. A total of 10 $\mu$l of 1:10 diluted PCR products were loaded onto the gel at starting positions at the following temperatures: 43°C for fragments I and III, 48°C for fragment II and 46°C for fragment IV. Gels were run at 6 W (10 V $cm^{-1}$) at 20°C for 15 min to allow entering of the samples into the gel at native conditions. Then, a temperature gradient ($T1 = 30$°C and $T2 = 70$°C) was superimposed on the gel parallel to the electric field. Running times at 7.5 W (8 V $cm^{-1}$) were 30 min. After electrophoresis, the gels were silver stained.

### *Microdissection and analysis of microsatellite polymorphisms*

To enrich for tumour cells, 8 $\mu$m sections of formalin-fixed paraffin-embedded tumours were subjected to microdissection as described (Speiser *et al.*, 1996).

Microsatellite analysis was performed for highly polymorphic dinucleotide repeat polymorphism at the loci *TP53* (Jones and Nakamura 1992) and D17S786 (Gyapay *et al.*, 1994) with primer and conditions described there.

### *RFLP analysis*

For the analysis of the intragenic RFLP (exon 4, *Bst*U1) a 259 bp fragment was amplified by PCR and analysed on a 4% (3/1 NuSieve/GTG-Agarose) agarose gel as described by Greenwald *et al.* (1992).

### *Immunohistochemistry*

Sections (3 $\mu$m) were cut, deparaffinised and fixed on poly-L-lysine-coated slides at room temperature. The sections were stained with high-affinity anti-p53 monoclonal antibody DO-1 (Vojtesek *et al.*, 1992) using the staining protocol previously described by Midgly *et al.* (1992) with the difference in the streptavidin–biotin step (Biogenex Super Sensitive system was used instead of Vector kit). 3,3-diaminobenzidine in 0.03% nickel sulphate was used as chromogen. In addition, a further three monoclonal antibodies to p53 protein, i.e. BP53.12, PAb 421, DO-7, and anti-p53 rabbit antiserum CM-1, were used in parallel for the comparisons but the results owing to minor difference in the staining intensity as compared with DO-1 are not presented in detail.

### *Fluorescence* in situ *hybridisation*

Dissociation of nuclei was as previously described (Sauter *et al.*, 1995*a*). The chromosome 17 centromere probe p17H8 was digoxigenated by nick translation. Cells on slides were denatured in 70% formamide/2 × sodium saline citrate (SSC) (1 × SSC is 0.15 M sodium chloride, 0.015 M sodium citrate), pH 7, at 75°C. The hybridisation mixture (10 $\mu$l) consisted of 30 ng of the centromere probe and 10 ng unlabelled sonicated herring sperm DNA in 55% formamide, 10% dextran sulphate and 2 × SSC (pH 7). After hybridisation the slides were washed in 55% formamide/2 × SSC, pH 7 at 45°C. Immunohistochemical probe detection using FITC-conjugated sheep anti-digoxigenin (Vector) and FITC-conjugated sheep anti-digoxigenin (Sigma) was described previously (Sauter *et al.*, 1995*a*). For each case, the centromere 17 count was scored in 100 cells. Monosomic cell counts below 10% do not indicate allelic loss, since this can also be found in normal tissue and is generally attributed to an inefficient hybridisation.

### Results

### *Patient characteristics*

The 52-year-old female patient had developed multiple primary malignancies (Table I), including liposarcoma of the left arm (T1) at age 40, Paget's disease and a concomitant intraductal carcinoma of the left breast (T2) at 42, Paget's disease and a concomitant ductal carcinoma *in situ* of the right breast (T3) at 45, two independent malignant histiocytomas of the biceps muscle of the left arm (T4) at 48 and of the pelvis (T5) at 49. One year later, when she was operated on for a pelvic recurrence of histiocytoma (T6), a metastasis of an adenocarcinoma of unknown origin was found in the omentum majus (T7).

### *Detection of a TP53 germ-line mutation*

To screen lymphocyte and tumour DNA for mutations in the *TP53* gene, regions spanning exons 5 to 8 were amplified by polymerase chain reaction (PCR). PCR products were subjected to TGGE analysis to detect bands with altered electrophoretic mobility. All tumours exhibited a band pattern that was in line with a possible mutation in exon 8. This pattern was also found in the DNA of the patient's peripheral blood lymphocytes (PBLs), indicating that the mutation was constitutional (Figure 1). All PCR products exhibiting this band pattern were directly sequenced. At position 2 in codon 278 a cytosine to thymine (C→T)

**Table I** LOH determined by microsatellite polymorphisms and TGGE; p53 immunohistochemistry expressed as staining intensity and percentage of positive nuclei

| | Loss of heterozygosity | | | p53 staining | Positive |
|---|---|---|---|---|---|
| *Tumours* | *TGGE* | *TP53* | *D17S768* | *Intensity* | *nuclei (%)* |
| T1 | + | + | + | Strong | >50% |
| T2 | – | + | – | Strong | >50% |
| T3 | – | – | – | Strong | >50% |
| T4 | + | + | + | ND | |
| T5 | + | + | + | Weak | 50% |
| T6 | + | + | + | Strong | 10% |
| T7 | – | – | – | Weak | <20% |

T1, liposarcoma, arm left; T2, breast cancer, left; T3, breast cancer, right; T4/5, maligant histiocytoma, arm left/pelvis; T6, pelvic recurrence of histiocytoma; T7, metastasis adenocarcinoma; Positive nuclei (%), percentage of p53-positive stained nuclei; +, LOH; –, no LOH; ND, not done.

**Figure 1** TGGE analysis of exon 8-specific PCR products of all family members analysed (**a**) and the various tumours of the affected patient. The affected patient and both her sons carry a point mutation resulting in denaturation of the PCR products at a lower melting temperature, whereas all other family members are characterised by wild-type homoduplex bands only. All tumours exhibited the same band pattern, the mutated homoduplex bands being dominant in tumours T1, T4, T5 and T6 indicating LOH affecting exon 8.

transition was detected, which resulted in an amino acid exchange from proline to leucine in the DNA-binding domain (Cho *et al*., 1994) of the p53 protein.

*p53 overexpression in the tumours*

The results of immunohistochemistry are shown in Table I. The p53-positive nuclear reaction was detected in all tumour specimens tested regardless of the histological type of the malignancy. Any positive staining was observed in the stroma.

*Family screening and haplotype analysis*

Exact construction of the patient's pedigree (Figure 2) did not reveal a high cancer incidence in the family history. The patient's mother had died of an unknown disease at age 50. One sister of her eight siblings had died of post-operative thromboembolism. DNA from PBLs of the patient's family was subjected to PCR–TGGE analysis of exon 8, which revealed that her daughter, five brothers, two sisters, father and aunt (maternal side) harboured wild-type alleles only. Both of her sons, however, displayed the same band pattern as their mother (Figure 1) and direct sequencing of the PCR products showed that they had inherited the mutant allele. To study haplotype associations (Figure 2), family members were typed using the microsatellite polymorphisms at or near *TP53* (D17S768, *TP53*) and an intragenic RFLP (exon 4, *Bst*U1) (data not shown). We were able to demonstrate that the defective allele originated from the maternal side because the patient's aunt, four of her brothers and one of her sisters had inherited the same allele, which however, was wild-type. The patient's two sons had also inherited this allele, but in the mutant form.

*Allelic imbalance in the tumours*

In four tumours we detected LOH both at *TP53* and at D17S768 (Table I, Figure 3). This was in line with the observation that on TGGE analysis the mutated homoduplex band was stronger than the wild-type homoduplex band (Figure 1), indicating that LOH directly affected the *TP53* gene. In tumour T2, LOH was found only at the microsatellite marker *TP53* (imbalance factor of 2.1). In tumours T3 and T7, no LOH was detectable. All tumour DNA analysed was derived from archival material and care was taken to maximise the number of tumour cells scraped off the slides for PCR analysis. LOH in each tumour involved loss of the allele inherited from the patient's father (Figure 3). The concordant results of TGGE and microsatellite analysis are a true indication of tumour DNA status and are not an artefact of PCR.

Furthermore, we applied FISH with a centromere probe to evaluate the chromosome 17 count. Tissue blocks from four tumours containing more than 60% tumour cells were considered adequate for FISH analyses. Only the local recurrence of the malignant pelvic histiocytoma (T6) showed a monosomic population of 41%, further corroborating the finding of LOH for this tumour. There was a considerable chromosome 17 heterogeneity on two of four tumours examined by FISH. The malignant pelvic histiocytoma (T5) itself contained two polysomic populations greater than 5% (chromosome 17 centromere count $n=3$: 19%, $n=4$: 55%) and the intraductal carcinoma (T2) four polysomic populations ($n=3$: 8%, $n=5$: 14%, $n=6$: 8%, $n>6$: 19%).

**Discussion**

In this study we report a constitutional point mutation at codon 278 in exon 8 of the *TP53* tumour-suppressor gene in a patient with multiple primary malignancies. This particular mutation has not yet been reported as a germ-line mutation. The mutant allele was also detected in the PBLs-DNA of the patient's two sons, but not in the other family members tested. The patient's aunt and five siblings carry this allele in a non-mutated form, which corroborates the hypothesis of a *de novo* mutation and suggests that the patient is the founder of a new cancer-prone family.

Codon 278 is not a mutational hot spot in sporadic cancer. Reviewing over 2500 cases of *TP53* mutations, Hollstein *et al*. (1994) found only 31 mutations at codon 278 and three C→T transitions at position 2 (Sameshima *et al*., 1992; Hollstein *et al*., 1990).

The C→T transition at position 2 is located in the DNA-binding domain (Cho *et al*., 1994) and results in an amino acid exchange from proline to leucine. It is likely that this exchange has an influence on the protein structure, since

**Figure 2** Pedigree. Circles represent females and squares represent males. Semi-solid symbols represent mutation carriers and dash is for deceased. The patient affected by multiple tumours (◐) is indicated by an arrow. Haplotypes were derived from analysis of polymorphic loci *TP53* and D17S768. The proband inherited allele A from her father and allele B from her mother; her aunt carrying the same allele B. The proband's mother's genotype is shown in parenthesis since it could not be analysed directly. It was reconstituted by allelotyping the patient's father, her aunt and seven siblings. Since allele B was found in non-mutated form in the aunt and five siblings, the *TP53* germ-line mutation is classified *de novo*. The mutated allele (**B**) was transmitted to the patient's two sons (◪) (age 20 and 23), who showed no evidence of disease at time of analysis.

**Figure 3** Analysis of polymorphic loci *TP53* (**a**) and D17S768 (**b**) indicating LOH at *TP53* for tumours T1, T2, T4, T5 and T6 and at D17S768 for T1, T4, T5 and T6. An allelic imbalance factor $>1.5$ calculated for the peaks in the tumour samples in relation to the peaks in the PBL-control (N) was chosen as an indication for LOH. Note that there are many shadow peaks that are thought to be most probably caused by erroneous nucleotide incorporation by *Taq* Polymerase, which may occur when the repeat unit is small, as is the case with the dinucleotide polymorphisms shown.

leucine is much more hydrophobic than proline and is more often found in the core region. This is the first report to demonstrate that this particular mutation alters the half-life of the protein. All tumour samples analysed showed positive staining on immunohistochemistry, excluding the possibility of a rare polymorphism. Although there are slight differences in the staining intensity and the proportion of p53-positive nuclei in the group of tumours examined (Table I), the positive uniformity and the substantial number of positive nuclei clearly indicate that the germ-line mutation in this case is associated with conformational changes in the p53 protein, its stabilization and potential loss of its function.

Five of the seven tumours showed LOH at and near the *TP53* locus as concordantly determined by microsatellite analysis and PCR–TGGE (Table I, Figures 1 and 3). This is in accordance with Knudson's two-hit hypothesis for the role of tumour-suppressor genes in neoplasia (Knudson, 1985). In the case of the pelvic recurrence of malignant histiocytoma (T6), the entire wild-type chromosome had been lost in approximately 40% of tumour cells as detected by FISH, explaining the allelic loss detected by TGGE and microsatellite analyses. In the intraductal carcinoma of the left breast (T2), LOH was found only at the *TP53* locus but not at the neighbouring locus D17S768, indicating that only a short DNA region was affected. However, the calculated imbalance factor for D17S768 was just slightly below the cut-off level for LOH, possibly indicating a sensitivity problem in detecting LOH for this locus in this tumour. This may either be caused by a low number of tumour cells in the tissue sample, or it may reflect the heterogeneity within a tumour, indicating that LOH may be a dynamic process during tumour progression. There is yet another indication that loss of the non-mutated allele is not the only fundamental basis for tumour development in this patient. In the Paget's disease accompanied by a ductal carcinoma *in situ* of the right breast (T3) and in the metastasis of an adenocarcinoma of unknown origin (T7), no LOH was detected by any of the methods used. However, positive immunohistochemical staining was seen in these tumour samples. It has previously been shown that in affected Li–Fraumeni family members a point mutation in *TP53* is insufficient for positive immunohistochemical staining in non-tumoral tissue (Eeles *et al.*, 1993; Malkin *et al.*, 1990). Therefore positive staining in the tumours without LOH cannot only be related to the point mutation, but also to a possible complexation of the wild-type p53 by a protein like MDM2 (Momand *et al.*, 1992;

Oliner *et al.*, 1992). Interference with such a protein would probably affect p53 activity, thus being the proposed 'second hit'. However, the nature of such another fundamental event apart from the point mutation is unclear at present.

The finding of a marked chromosome 17 heterogeneity with several separate polysomic populations in two tumours is consistent with the postulated role of p53 in terms of preserving genomic stability. This is in line with our findings that more than three polysomic populations occur almost exclusively in tumours with a p53 alteration (Sauter *et al.*, 1995*b*).

There is strong evidence that some germ-line *TP53* mutations are closely related to cancer development in their carriers, because in p53 families early cancer development is associated with the presence of the germ-line mutation. To this point, we have not yet been able to prove that cancer in our patient was indeed caused by the constitutional *TP53* mutation but we believe that there is ample evidence to support this notion: (1) the affected codon lies in a highly conserved domain: (2) the mutation leads to an amino acid exchange in the DNA-binding domain, resulting in a conformational change and in stabilisation of the protein; (3) the mutation seems to play a role in the development of sporadic cancer; (4) frequent loss of the wild-type allele in the tumours has been demonstrated; and (5) the mutant allele coincides with the development of multiple primary cancers.

Reviewing the spectrum of tumours and the age of onset that were reported in *TP53* germ-line mutation carriers, Birch *et al.* (1994) separated classical Li–Fraumeni syndrome families from families exhibiting some but not all of the features of Li–Fraumeni syndrome. In classical Li–Fraumeni syndrome families, *TP53* germ-line mutations are much more common than in other cancer-prone families. As to the histological type of her tumours, our patient fits into both groups described. The future family history will decide to which of the two groups they actually belong.

**Acknowledgements**
We are grateful to Dr Joe W Gray (Director of the UCSF Resource for Molecular Genetics), who has generously supplied the p17H8 probe (chromosome 17 centromere); we are also grateful to Ms M Hedvika (University, Bern) and Mr L Szabo (University, Vienna) and Mr R Kurzbauer (Institute of Molecular Pathology, Vienna) for technical assistance. This work was supported by the 'Medizinish-Wissenschaftlicher Fonds des Bürgermeisters der Bundeshauptstadt Wien' and the Grant Agency of the Czech Republic (grant no. 312/93/2329).

### References

BIRCH JM, HARTLEY AL, TRICKER KJ, PROSSER J, CONDIE A, KELSEY AM, HARRIS M, JONES PH, BINCHY A, CROWTHER D, CROWTHER D, CRAFT AW, EDEN OB, EVANS GR, THOMPSON E, MANN JR, MARTIN J, MITCHELL LD AND SANTIBANEZ-KOREF MF. (1994). Prevalence and diversity of constitutional mutations in the p53 gene among 21 Li-Fraumeni families. *Cancer Res.*, **54**, 1298–1304.

CARON DE FROMENTEL C AND SOUSSI T. (1992). TP53 tumor suppressor gene: a model for investigating human mutagenesis. *Genes Chrom. Cancer*, **4**, 1–15.

CHO Y, GORINA S, JEFFREY PD AND PAVLETICH NP. (1994). Crystal structure of a p53 tumor suppressor-DNA complex: understanding tumorigenic mutations. *Science*, **265**, 346–355.

EELES RA, WARREN W, KNEE G, BARTEK J, AVERILL D, STRATTON MR, BLAKE PR, TAIT DM, LANE DP, EASTON DF, YARNOLD JR, COOPER CS AND SLOANE JP. (1993). Constitutional mutation in exon 8 of the p53 gene in a patient with multiple primary tumours: molecular and immunohistochemical findings. *Oncogene*, **8**, 1269–1276.

GREENWALD BD, HARPAZ N, YIN J, HUANG Y, TONG Y, BROWN VL, MCDANIEL T, NEWKIRK C, RESAU JH AND MELTZER SJ. (1992). Loss of heterozygosity affecting the p53, Rb, and mcc/apc tumor suppressor gene loci in dysplastic and cancerous ulcerative colitis. *Cancer Res.*, **52**, 741–745.

GYAPAY G, MORISSETTE J, VIGNAL A, DIB C, FIZAMES C, MILLAUSSEAU P, MARC S, BERNARDI G, LATHROP M AND WEISSENBACH J. (1994). The 1993–94 Genethon human genetic linkage map. *Nature Genet.*, **7**, 246–339.

HOLLSTEIN M, RICE K, GREENBLATT MS, SOUSSI T, FUCHS R, SORLIE T, HOVIG E, SMITH SORENSEN B, MONTESANO R AND HARRIS CC. (1994). Database of p53 gene somatic mutations in human tumors and cell lines. *Nucleic Acids Res.*, **12**, 3551–3555.

HOLLSTEIN MC, METCALF RA, WELSH JA, MONTESANO R AND HARRIS CC. (1990). Frequent mutation of the p53 gene in human esophageal cancer. *Proc. Natl Acad. Sci. USA*, **87**, 9958–9961.

JONES MH AND NAKAMURA Y. (1992). Detection of loss of heterozygosity at the human TP53 locus using a dinucleotide repeat polymorphism. *Genes Chrom. Cancer*, **5**, 89–90.

KNUDSON AG. (1985). Hereditary cancer, oncogenes, and antioncogenes. *Cancer Res.*, **45**, 1437–1443.

LANE DP. (1992). p53, guardian of the genome. *Nature*, **358**, 15–16.

LEVINE AJ, MOMAND J AND FINLAY CA. (1991). The p53 tumour suppressor gene. *Nature*, **351**, 453–456.

LI FP AND FRAUMENI JFJ. (1969). Soft-tissue sarcomas, breast cancer, and other neoplasms. A familial syndrome? *Ann. Intern. Med.*, **71**, 747–752.

MALKIN D, LI FP, STRONG LC, FRAUMENI JF Jr, NELSON CE, KIM DH, KASSEL J, GRYKA MA, BISCHOFF FZ, TAINSKY M AND FRIEND SH. (1990). Germ line p53 mutations in a familial syndrome of breast cancer, sarcomas, and other neoplasms. *Science*, **250**, 1233–1238.

MIDGLEY CA, FISHER CJ, BARTEK J, VOJTESEK B, LANE D AND BARNES DM. (1992). Analysis of p53 expression in human tumours: an antibody raised against human p53 expressed in *Escherichia coli*. *J. Cell. Sci.*, **101**, 183–189.

MOMAND J, ZAMBETTI GP, OLSON DC, GEORGE D AND LEVINE, AJ. (1992). The mdm-2 oncogene product forms a complex with the p53 protein and inhibits p53-mediated transactivation. *Cell*, **69**, 1237–1245.

OLINER JD, KINZLER KW, MELTZER PS, GEORGE DL AND VOGELSTEIN, B. (1992). Amplification of a gene encoding a p53-associated protein in human sarcomas. *Nature*, **358**, 80–83.

SAMESHIMA Y, MATSUNO Y, HIROHASHI S, SHIMOSATO Y, MIZOGUCHI H, SUGIMURA T, TERADA M AND YOKOTA J. (1992). Alterations of the p53 gene are common and critical events for the maintenance of malignant phenotypes in small-cell lung carcinoma. *Oncogene*, **7**, 451–457.

SAUTER G, MOCH H, CARROLL P, KERSCHMANN R, MIHATSCH MJ AND WALDMANN FW. (1995*a*). Chromosome-9 loss detected by fluorescence *in situ* hybridisation in bladder cancer. *Int. J. Cancer*, **64**, 99–103.

SAUTER G, MOCH H, GASSER TH, MIHATSCH MJ AND WALDMAN FM. (1995*b*). Heterogeneity of chromosome 17 and erbB-2 gene copy number in primary and metastatic bladder cancer. *Cytometry*, **21**, 40–46.

SHEFFIELD VC, COX DR, LERMAN LS AND MYERS RM. (1989). Attachment of a 40-base-pair G + C-rich sequence (GC-clamp) to genomic DNA fragment by the polymerase chain reaction results in improved detection of single-base changes. *Proc. Natl Acad. Sci. USA*, **86**, 232–236.

SOUSSI T, CARON DE FROMENTEL C AND MAY P. (1990). Structural aspects of the p53 protein in relation to gene evolution. *Oncogene*, **5**, 945–952.

SPEISER P, GHAREHBAGHI-SCHNELL E, SCHNEEBERGER C, EDER S AND ZEILLINGER R. (1996). Microdissection as a means to verify allelic imbalance in tumor biopsy samples. *Anticancer Res.*, **16**, 461–464.

SRIVASTAVA S, ZOU ZQ, PIROLLO K, BLATTNER W AND CHANG EH. (1990). Germ-line transmission of a mutated p53 gene in a cancer-prone family with Li-Fraumeni syndrome. *Nature*, **348**, 747–749.

VOGELSTEIN B AND KINZLER KW. (1992). p53 function and dysfunction. *Cell*, **70**, 523–526.

VOJTESEK B, BARTEK J, MIDGLEY CA AND LANE DP. (1992). An immunochemical analysis of the human nuclear phosphoprotein p53. New monoclonal antibodies and epitope mapping using recombinant p53. *J. Immunol. Methods*, **151**, 237–244.

ZAKUT HOURI R, BIENZ TADMOR B, GIVOL D AND OREN M. (1985). Human p53 cellular tumor antigen: cDNA sequence and expression in COS cells. *EMBO J.*, **4**, 1251–1255.